# Analysis of the Genomic Sequence of *ABO* Allele Using Next-Generation Sequencing Method

**DOI:** 10.3389/fimmu.2022.814263

**Published:** 2022-07-06

**Authors:** Yanmin He, Xiaozhen Hong, Jingjing Zhang, Ji He, Faming Zhu, He Huang

**Affiliations:** ^1^ Bone Marrow Transplantation Center, The First Affiliated Hospital, Zhejiang University School of Medicine, Hangzhou, China; ^2^ Institute of Transfusion medicine, Blood Center of Zhejiang Province, Hangzhou, China; ^3^ Key Laboratory of Blood Safety Research of Zhejiang Province, Hangzhou, China; ^4^ Liangzhu Laboratory, Zhejiang University Medical Center, Hangzhou, China; ^5^ Institute of Hematology, Zhejiang University, Hangzhou, China; ^6^ Zhejiang Province Engineering Laboratory for Stem Cell and Immunity Therapy, Hangzhou, China

**Keywords:** ABO subtypes, next-generation sequencing, single nucleotide variant, allele recombination, an erythroid cell-specific regulatory element

## Abstract

**Background:**

Although many molecular diagnostic methods have been used for *ABO* genotyping, there are few reports on the full-length genomic sequence analysis of the *ABO* gene. Recently, next-generation sequencing (NGS) has been shown to provide fast and high-throughput results and is widely used in the clinical laboratory. Here, we established an NGS method for analyzing the sequence of the start codon to the stop codon in the *ABO* gene.

**Study Design and Methods:**

Two pairs of primers covering the partial 5’-untranslated region (UTR) to 3’-UTR of the *ABO* gene were designed. The sequences covering from the start codon to the stop codon of the *ABO* gene were amplified using these primers, and an NGS method based on the overlap amplicon was developed. A total of 110 individuals, including 88 blood donors with normal phenotypes and 22 ABO subtypes, were recruited and analyzed. All these specimens were first detected by serological tests and then determined by polymerase chain reaction sequence-based typing (PCR-SBT) and NGS. The sequences, including all the intron regions for the specimens, were analyzed by bioinformatics software.

**Results:**

Among the 88 blood donors with a normal phenotype, 48 homozygous individuals, 39 heterozygous individuals, and one individual with a novel *O* allele were found according to the results of the PCR-SBT method. Some single-nucleotide variants (SNV) in intronic regions were found to be specific for different *ABO* alleles from 48 homozygous individuals using the NGS method. Sequences in the coding region of all specimens using the NGS method were the same as those of the PCR-SBT method. Three intronic SNVs were found to be associated with the ABO subtypes, including one novel intronic SNV (c.28+5956T>A). Moreover, six specimens were found to exhibit DNA recombination.

**Conclusion:**

An NGS method was established to analyze the sequence from the start codon to the stop codon of the *ABO* gene. Two novel *ABO* alleles were identified, and DNA recombination was found to exist in the *ABO* alleles.

## Introduction

ABO is the most important blood group system in clinical transfusion medicine (1). The *ABO* gene is located on chromosome 19 and is approximately 20 kb from the start codon to the stop codon, including a 1062 bp coding region and various length intronic regions. Many molecular diagnostic tests have been developed to identify *ABO* alleles (2–11). To date, more than 300 different *ABO* alleles have been characterized (12). However, the sequence from the start codon to the stop codon of the *ABO* gene, including intronic regions, has rarely been reported.

Polymerase chain reaction specific sequence primer (PCR-SSP) (13), PCR sequence-based typing (PCR-SBT) (10, 14–18), gene-chip (19, 20), and next-generation sequencing (NGS) methods (21–24) have been used for *ABO* genotyping. The PCR-SBT method has advantages in finding new variants. Most of the current PCR-SBT methods focus on the sequencing of exons 6 and 7 of the *ABO* gene, which is the most polymorphic coding region in the *ABO* gene. A method for sequencing exons 1 to 7 of the *ABO* gene by the PCR-SBT method was also established, which has been used to discriminate some ABO subtypes (18). However, no variants have been found in the exonic regions and splice sites in some ABO subtypes, indicating that certain variants may exist in other regions of the *ABO* gene. Takahashi et al. developed a long-range PCR (LR-PCR) with peptide nucleic acid (PNA) technology for *ABO* genotyping and found a single nucleotide variant (SNV) in intron 1 associated with the A_m_ subtype by direction sequencing (25). Therefore, it is necessary to establish an alternative method for analyzing the full-length sequences of the *ABO* gene.

The development of NGS has changed the landscape of molecular diagnostic testing, and this method is widely used in the clinical laboratory field due to its fast and high-throughput properties (21–24). Fichou et al. reported the application of the NGS method for red blood cell (RBC) genotyping by the Ion Torrent platform in 2014 (21). In addition, a study from the German marrow donor center revealed *ABO* allele frequencies based on the sequence of exons 6 and 7 by the NGS method (22). Wu et al. reported resolving heterogeneity in donors with serology discrepancies using targeted NGS (23). Moreover, NGS technology has been used in other systems, such as JK, KEL, and FY (26) analysis. Recently, Tounsi et al. used LR-PCR with NGS to obtain the complete sequences of *RHD* genes (27). Our lab reported a new method for analyzing the full genomic sequence from the start codon to the stop codon of the *ABO* gene and found six splice site variations (28). In this study, we detected various specimens from blood donors and patients by the LR-PCR NGS method.

## Materials and Methods

### Specimen Collection and Study Design

Eighty-eight individuals with different ABO group phenotypes were selected from voluntary blood donors in the Blood Center of Zhejiang Province, China. In addition, 22 ABO subtypes were analyzed, which were previously collected from blood donors in the Blood Center of Zhejiang Province or from patients in the hospitals of Hangzhou City, China. Peripheral blood specimens were collected in 5 ml tubes with EDTA anticoagulant for serological testing and molecular diagnosis. This study was approved by the Ethical Scientific Committee of Zhejiang Provincial Blood Center, China. Informed consent was obtained from all participants.

#### Serological Analysis

#### Four Common ABO Phenotypes in 88 Individuals

The ABO forward and reverse grouping for 88 blood donors was performed by a microplate test on an automatic analyzer (PK7300, Beckman Coulter, Inc., S. Kraemer Boulevard Brea, CA, USA) using monoclonal anti-A and anti-B reagents (Shanghai Hemo-pharmaceutical & Biological, Shanghai, China) and A and B red cells according to our previous reports (18, 29).

#### Serological Analysis for ABO Subtypes

These individuals were initially subjected to ABO grouping according to our previous reports (18, 29). All variant samples were found with ABO serological grouping discrepancies, and anti-A, anti-B, anti-A_1_, anti-A,B, and anti-H were added to test in tubes for ABO-related antigens. If necessary, the adsorption-elution test was used to detect weak antigens as documented in the AABB technical manual (30). ABO subtypes were classified according to serological characteristics (30).

### Genomic DNA Extraction

Genomic DNA (gDNA) was extracted from peripheral blood specimens using an automatic nucleic acid extraction instrument according to the manufacturer’s instructions (Roche Diagnostics Inc., Shanghai, China). The optical density ratio of DNA was determined by a spectrophotometer (NanoDrop, Thermo Fisher Scientific, Inc., Shanghai, China). A final concentration of 30 ng/μl gDNA was prepared and stored in Tris-EDTA buffer (Roche Diagnostics Inc., Shanghai, China) for further experiments.

### Sequencing the Coding Region of the *ABO* Gene With the PCR-SBT Method

The coding region sequence of the *ABO* gene was analyzed as previously described (29). Briefly, exons 1 to 7 of the *ABO* gene were amplified and sequenced bidirectionally using a Bigdye Terminator Cycle v3.1 Sequencing kit (Applied Biosystems, Foster City, CA, USA). The sequence data were analyzed by Seqscape 2.5 software (Applied Biosystems, Foster City, CA, USA) and assigned for the *ABO* allele according to the nucleotide sequence of the polymorphic position based on the standard of the red cell immunogenetics and blood group terminology of the International Society of Blood Transfusion (ISBT).

### Analysis of the Sequence From the Start Codon to the Stop Codon of the *ABO* Gene Using Next-Generation Sequencing

**Figure 1:**
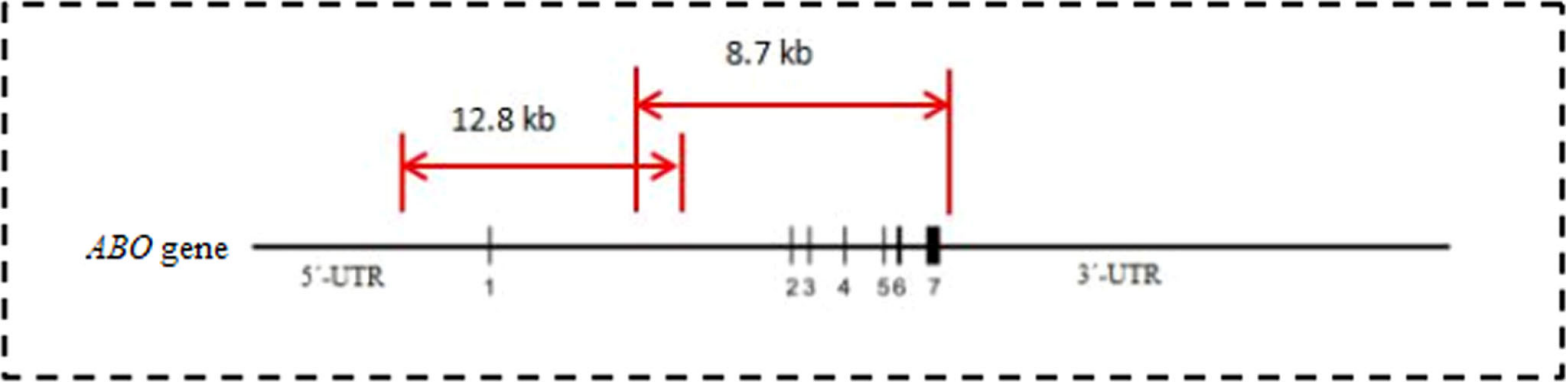
Amplify the sequence of the *ABO* gene by LR-PCR technology. Two pairs of primers with the coverage from 5’-UTR to 3’-UTR of the *ABO* gene were designed. Two overlap amplicons with the length of 12.8 kb and 8.7 kb were amplified respectively. The schematic drawing of ABO gene cited from reference [34].

Two pairs of primers were designed according to the sequence of the *ABO* gene (GenBank ID: NG_006669.2). The primer sequences are listed in **Table 1**. The overlapping amplicons (12.8 kb and 8.7 kb) from the primer pairs covered the sequence from the start codon to the stop codon (**Figure 1**). In brief, the sequence of the *ABO* gene was first amplified by a long fragment amplification technique. All PCRs were optimized by performing in 25 μl volumes containing approximately 100 ng of genomic DNA in 5×GXL PCR buffer, 0.5 μmol/L of each primer, 200 μmol/L of each dNTP, 2.0 mmol/L MgCl_2,_ and 0.625 units of GXL-Taq DNA polymerase (TaKaRa, Dalian, China). The amplification was performed on an ABI PCR 9700 instrument (Applied Biosystems). After purification by the Agencourt AMPure XP (Beckman Coulter Inc., Carlsbad, CA, USA) according to the manufacturer’s instructions, the two amplicons were quantified using the Qubit double-stranded DNA High Sensitivity assay kit (Life Technologies, Shanghai, China) to create an equimolar pool, which ensured an equal depth of coverage across the *ABO* gene. The library for the amplicon was prepared with a TransNGS Tn5 DNA library Prep Kit (TransGen Biotech Inc., Beijing, China) and was sequenced with MiSeq reagent cartridge v2 for 300 cycles (Illumina, Inc., San Diego, CA, USA). The reagent cartridge and flow cell were placed on the Illumina MiSeq for cluster generation and 2 ×150 bp paired-end sequencing. All the sequencing data in FASTQ format were analyzed by CLC Genomics workbench 20.0 (Qiagen, Shanghai, China) with default settings, mapping to the reference of ABO*A.01.01 (GenBank ID: NG_006669.2), and the genotypes of the specimens were assigned according to the nucleotide sequences. *ABO* variant detection was performed on a minimum coverage of 50 and analyzed on a single-base basis considering different parameters, including number and percentage of reads and nucleotide count. The average coverage for partial samples is shown in **Supplementary S1**.

**Table 1:** Oligonucleotide primers used for *ABO* LR-PCR amplification.

Primer name	Sequence (5´-3´)	Coverage	Size, kb	PCR parameter
ABO1 longF	GCTTCCAGCTTTTGGCTATG	5´-UTR~Intron 1	12.8	94°C,1 min/ 30 cycles for 98°C,10 s; 68°C, 10 min
ABO1 longR	GTGACCACGGAGCGATTTAT			
ABOe27longF	GGATTAACAATGGCGTGCTT	Intron 1~3´-UTR	8.7	
ABOe27longR	GGACGGACAAAGGAAACAGA			

## Results

### The Coding Sequence of the 88 Individuals With Four Common ABO Phenotypes and a Novel *O* Allele Were Identified

In total, 29 group A, 26 group B, 28 group O, and five group AB blood donors were chosen (**Table 2**), which were not randomly collected from the blood donors. In the serological typing, the results of ABO forward grouping were consistent with the reverse grouping for all samples. The genotypes of these samples are listed in **Table 2**, including 48 homozygous samples (the numbers of ABO*A1.02/ABO*A1.02, ABO*B.01/ABO*B.01, ABO*O.01.01/ABO*O.01.01, and ABO*O.01.02/ABO*O.01.02 were 12, 15, 13 and 8, respectively), 39 heterozygous samples (the numbers of ABO*A1.02/ABO*O.01.01, ABO*A1.02/ABO*O.01.02, ABO*A1.01/ABO*A1.02, ABO*A1.01/ABO*O.01.01, ABO*B.01/ABO*O.01.01, ABO*B.01/ABO*O.01.02, ABO*O.01.01/ABO*O.01.02, and ABO*A1.02/ABO*B.01 were 8, 6, 1, 2, 5, 6, 6, and 5, respectively) and one individual with one ABO*O.01.01 allele. Compared to the ABO*O.01.01 allele, the novel ABO*O allele has a G>A variant at position 964 of exon 7, which could not change the amino acids due to the existence of c.261delG (resulting in p. Thr88Profs*31) in the ABO*O.01.01 allele. The nucleotide sequence of this novel allele has been submitted to GenBank (accession number OL348386).

**Table 2:** The results for 88 individuals with four common ABO phenotypes.

Group^#^	ABO Serology					specimen Number	Genotyping by Sanger sequencing	Genotyping by NGS
		Forward		Reverse				
	Phenotype	Anti-A	Anti-B	A cell	B Cell			
1	A	4+	0	0	3+~4+	12	ABO*A1.02/ABO*A1.02	V
	B	0	4+	3+~4+	0	14	ABO*B.01/ABO*B.01	V
	B	0	4+	3+~4+	0	1	ABO*B.01/ABO*B.01 ^£^	V
	O	0	0	3+~4+	3+~4+	13	ABO*O.01.01/ABO*O.01.01	V
	O	0	0	3+~4+	3+~4+	8	ABO*O.01.02/ABO*O.01.02	V
2	O	0	0	3+~4+	3+~4+	1	ABO*O.01.01/ABO*O.01.01(c.964G>A) ^&^	V
	A	4+	0	0	3+~4+	8	ABO*A1.02/ABO*O.01.01	V
	A	4+	0	0	3+~4+	6	ABO*A1.02/ABO*O.01.02	V
	A	4+	0	0	3+~4+	1	ABO*A1.01/ABO*A1.02	V
	A	4+	0	0	3+~4+	2	ABO*A1.01/ABO*O.01.01	V
	B	0	4+	3+~4+	0	5	ABO*B.01/ABO*O.01.01	V
	B	0	4+	3+~4+	0	6	ABO*B.01/ABO*O.01.02	V
	O	0	0	3+~4+	3+~4+	6	ABO*O.01.01/ABO*O.01.02	V
	AB	4+	4+	0	0	5	ABO*A1.02/ABO*B.01	V

V indicates the results of the ABO genotype for the specimens by NGS method were consistent with those of PCR-SBT. ^#^88 individuals were divided into two groups; group 1 contained 48 homozygous individuals; group 2 contained an individual with a novel O allele and 39 heterozygous individuals. ^£^allele recombination was found in this individual. ^&^The novel allele was identified in this individual. *ABO allele nomenclature.

### Some SNVs in Intronic Regions Were Specific for Different *ABO* Alleles Using NGS

To explore the polymorphism of some SNVs in the intronic region, the sequences of the 48 individuals with homozygotes were first analyzed using the NGS method. Compared to the sequence of ABO*A1.01, the number of polymorphism sites in the intronic region was 4, 32, 97, and 113 in the 12 individuals with ABO*A1.02/ABO*A1.02, 14 individuals with ABO*B.01/ABO*B.01, 13 individuals with ABO*O.01.01/ABO*O.01.01, and eight individuals with ABO*O.01.02/ABO*O.01.02, respectively; see the details in **Supplementary S2**. However, one individual with the ABO*B.01/ABO*B.01 genotype was found to exhibit allele recombination, which is described in the subsequent section in detail.

Some SNVs were specific for different *ABO* alleles. Twelve, 10, and 35 SNVs were associated with ABO*B.01, ABO*O.01.01, and ABO*O.01.02, respectively, which are listed in **Table 3**. The raw data for all the intronic SNVs are also shown in **Supplementary S2**. All of these specific intronic SNVs were also confirmed in 39 other heterozygous specimens by NGS.

**Table 3:** The Nucleotide change of intronic sequences for different *ABO* allotypes.

Group	genotype	Nucleotide change	Location	Group	genotype	Nucleotide change	Location
1	ABO*B.01/ABO*B.01	c.28+4282A>G	Intron 1	3	ABO*O.01.02/ ABO*O.01.02	c.156-389A>G	Intron 3
		c.28+6120T>C	Intron 1			c.156-208C>T	Intron 3
		c.29-4732T>G	Intron 1			c.156-174T>C	Intron 3
		c.29-4604G>A	Intron 1			c.156-95C>T	Intron 3
		c.29-3924C>T	Intron 1			c.203+28G>C	Intron 4
		c.29-86G>A	Intron 1			c.203+72_203+73insGTGTGGACAGAAG	Intron 4
		c.240-25A>G	Intron 5			c.203+114C>T	Intron 4
		c.374+42G>T	Intron 6			c.203+163G>A	Intron 4
		c.374+271A>G	Intron 6			c.203+215_203+216delinsGC	Intron 4
		c.374+280C>T	Intron 6			c.203+346T>G	Intron 4
		c.375-425A>G	Intron 6			c.203+738T>G	Intron 4
		c.375-152G>A	Intron6			c.204-511C>T	Intron 4
2	ABO*O.01.01/ABO*O.01.01	c.29-780delinsGG	Intron 1			c.204-220G>A	Intron 4
		c.29-746T>C	Intron 1			c.204-191T>C	Intron 4
		c.29-658G>A	Intron 1			c.204-176T>G	Intron 4
		c.98+362C>T	Intron 1			c.204-61dupC	Intron 4
		c.99-186C>A	Intron 1			c.239+103_239+105insC[3]	Intron 5
		c.155+575C>T	Intron 3			c.240-249C>T	Intron 5
		c.204-9T>C	Intron 4			c.240-105C>A	Intron 5
		c.240-219G>A	Intron 5			c.240-28G>A	Intron 5
		c.374+163C>T	Intron 6			c.374+89T>A	Intron 6
		c.375-269G>A	Intron 6			c.374+188G>A	Intron 6
3	ABO*O.01.02/ABO*O.01.02	c.28+175C>T	Intron 1			c.374+226C>T	Intron 6
		c.29-554A>C	Intron 1			c.374+235C>G	Intron 6
		c.29-286A>C	Intron 1			c.374+493T>C	Intron 6
		c.155+205C>T	Intron 3			c.375-336G>A	Intron 6
		c.155+479C>T	Intron 3			c.375-42A>G	Intron 6
		c.155+525A>T	Intron 3			c.375-40G>A	Intron 6
		c.156-483T>C	Intron 3				

*ABO allele nomenclature.

### The Ability of the NGS Method for Variant Identification in the Coding Region Was the Same as That of the PCR-SBT Method

Among the 22 ABO subtypes, 17 individuals with variations in the coding region have been previously found using the PCR-SBT method (**Table 4**). All variation sites in exonic regions of these specimens were also detected using the NGS method. Among them, two A_3_ specimens showed a deletion of G at position 106 in exon 3 (16), and four B_3_ specimens and one AB_3_ specimen showed a G>A variant at position 28 in exon 1. The variants of other specimens were located in exon 7, including 389T>C (18), 410C>T (17), 467C>T, 539G>C (7), 541T>C (9), 700C>G (4), 701C>T (12), 721C>T, 803G>C (2), and 940A>G (11).

**Table 4:** The results of the 17 ABO subtypes with variations in the coding region by Sanger sequencing and NGS method.

Sample ID	ABO Serology								genotype	Nucleotide change	Amino acid change	Location
	Phenotype	Forward					Reverse					
		Anti-A	Anti-B	Anti-A,B	Anti-A1	Anti-H	A cell	B cell				
19001	A_3_	mf	0	mf	0	3+	0	4+	ABO*A1.02/ABO*O.01.01	c.106delG^[16]^	p.Val36Serfs*37	Exon 3
19014	A_3_	mf	0	mf	±	4+	0	4+	ABO*A1.02/ABO*O.01.02	c.106delG^[16]^	p.Val36Serfs*37	Exon 3
19007	A_w_	±	0	±	0	4+	1+	4+	ABO*A1.02/ABO*O.01.01	c.389T>C^[18]^	p.Leu130Pro	Exon 7
19046	Ael	el(1+)	0	0	0	4+	2+	4+	ABO*A1.02/ABO*O.01.01	c.410C>T^[17]^	p.Ala137Val	Exon 7
19035	A_w_B	1+	4+	4+	0	4+	1+	0	ABO*AW.37/ABO*B.01	c.940A>G^[11]^	p.Lys314Glu	Exon 7
19041	B_w_	0	1+	1+	0	4+	4+	1+	ABO*BW.03/ABO*O.01.02	c.721C>T^[6]^	p.Arg241Trp	Exon 7
19005	B_w_	0	mf	mf	0	4+	4+	1+	ABO*BW.28/ABO*O.01.01	c.541T>C^[9]^	p.Trp181Arg	Exon 7
19026	A_2_B	2+	4+	4+	0	3+	2+	0	ABO*A2.08/ABO*B.01	c.467C>T, c.539G>C^[8]^	p.Pro156Leu; p.Arg180Pro	Exon 7
19039	A_2_B	1+	4+	4+	0	2+	1+	0	ABO*A2.07/ABO*B.01	c.539G>C^[7]^	p.Arg180Pro	Exon 7
19011	A_w_B	2+	4+	4+	0	4+	2+	0	ABO*BA.02/ABO*O.01.02	c.700C>G^[4]^	p.Leu266Met	Exon 7
19019	A_w_B	1+	3+	3+	0	2+	1+	0	ABO*BA.07/ABO*O.01.02	c.701C>T^[12]^	p.Pro234Leu	Exon 7
19034	AB_w_	4+	2+	4+	0	4+	0	1+	ABO*cisAB.01/ABO*O.01.02	c.467C>T, c.803G>C^[2]^	p.Pro156Leu; p.Gly268Ala	Exon 7
19048^#^	B_3_	0	mf	mf	0	4+	4+	0	ABO*B3.10/ABO*O.01.02	c.28G>A^[42]^	p.Gly10Arg	Exon 1
19008^#^	B_3_	0	mf	mf	0	4+	4+	0	ABO*B3.10/ABO*O.01.02	c.28G>A	p.Gly10Arg	Exon 1
19047^#^	B_3_	0	mf	mf	0	4+	4+	0	ABO*B3.10/ABO*O.01.01	c.28G>A	p.Gly10Arg	Exon 1
19004^#^	B_3_	0	mf	mf	0	4+	3+	0	ABO*B3.10/ABO*O.01.01	c.28G>A	p.Gly10Arg	Exon 1
19032^#^	AB_3_	4+	±	4+	4+	4+	0	0	ABO*A1.02/ABO*B3.10	c.28G>A	p.Gly10Arg	Exon 1

^#^Allele recombination was found in the five ABO subtypes by NGS method. mf, mixed field. el, adsorption-elution test. 0 means no agglutination. *ABO allele nomenclature.

### One Novel Intronic SNV for ABO Subtype Specimens Was Identified

**Figure 2:**
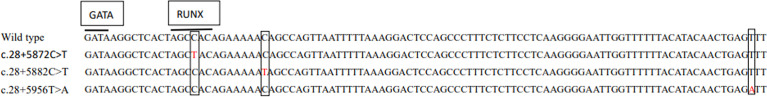
Three variations are located in the first intron of the *ABO* gene. The wild-type sequence between c.28+5856 and c.28+5958 in Intron 1 of the *ABO* is shown at the top. The motifs for GATA and RUNX1 are indicated by overbars. In alignment with the wild-type sequence, three variants are shown in red.

Among the 22 ABO subtypes, five individuals lacked variation in the exons or splice sites by PCR-SBT in the previous study (**Table 5**). However, some variations were found in an erythroid cell-specific regulatory element in intron 1 using the NGS method in these individuals. As shown in **Figure 2**, c.28+5872C>T was found in two specimens with the B_3_ and AB_3_ phenotypes, c.28+5882C>T was found in two specimens with the B_weak_ and AB_weak_ phenotypes, and c.28+5956T>A was found in the specimen with the A_3_ phenotype (**Table 5**). c.28+5956T>A was a novel intronic SNV and was first found in the ABO variant. Further, this novel SNV was found on the same sequencing read that does not contain SNVs for the B or the O allele, indicating it could be assigned on A allele. The locations of the three variations in the first intron 1 sequence are shown in **Figure 2**. The SNVs in the intronic region for five individuals are shown in **Supplementary S3**.

**Table 5:** The results of the five ABO subtypes with no variations in the coding region by NGS method.

Sample ID	Phenotype	ABO Serology							Genotype	Nucleotide change	GenBank ID	Location
		Forward					Reverse					
		Anti-A	Anti-B	Anti-A,B	Anti-A1	Anti-H	Acell	Bcell				
19053	A_3_	mf	0	mf	mf	4+	0	4+	ABO*A1.02/ABO*O.01.02	c.28+5956T>A^#^	OL339339	Intron 1
18121	B_3_	0	mf	mf	0	3+	3+	0	ABO*B.01/ABO*O.01.01	c.28+5872C>T^#^	OL339341	Intron 1
18103	AB_3_	4+	mf	4+	4+	3+	0	0	ABO*A1.02/ABO*B.01	c.28+5872C>T^#^	OL339341	Intron 1
19010	B_w_	0	3+	3+	0	4+	4+	0	ABO*B.01/ABO*O.01.01	c.28+5882C>T^#^	OL339342	Intron 1
19013	AB_w_	4+	1+	4+	4+	3+	0	0	ABO*A1.02/ABO*B.01	c.28+5882C>T^#^	OL339342	Intron 1

^#^All the variants in this Table were described according to the reference sequence of NG_006669.2.

### Allele Recombination Was Found in the ABO Specimens

**Figure 3:**
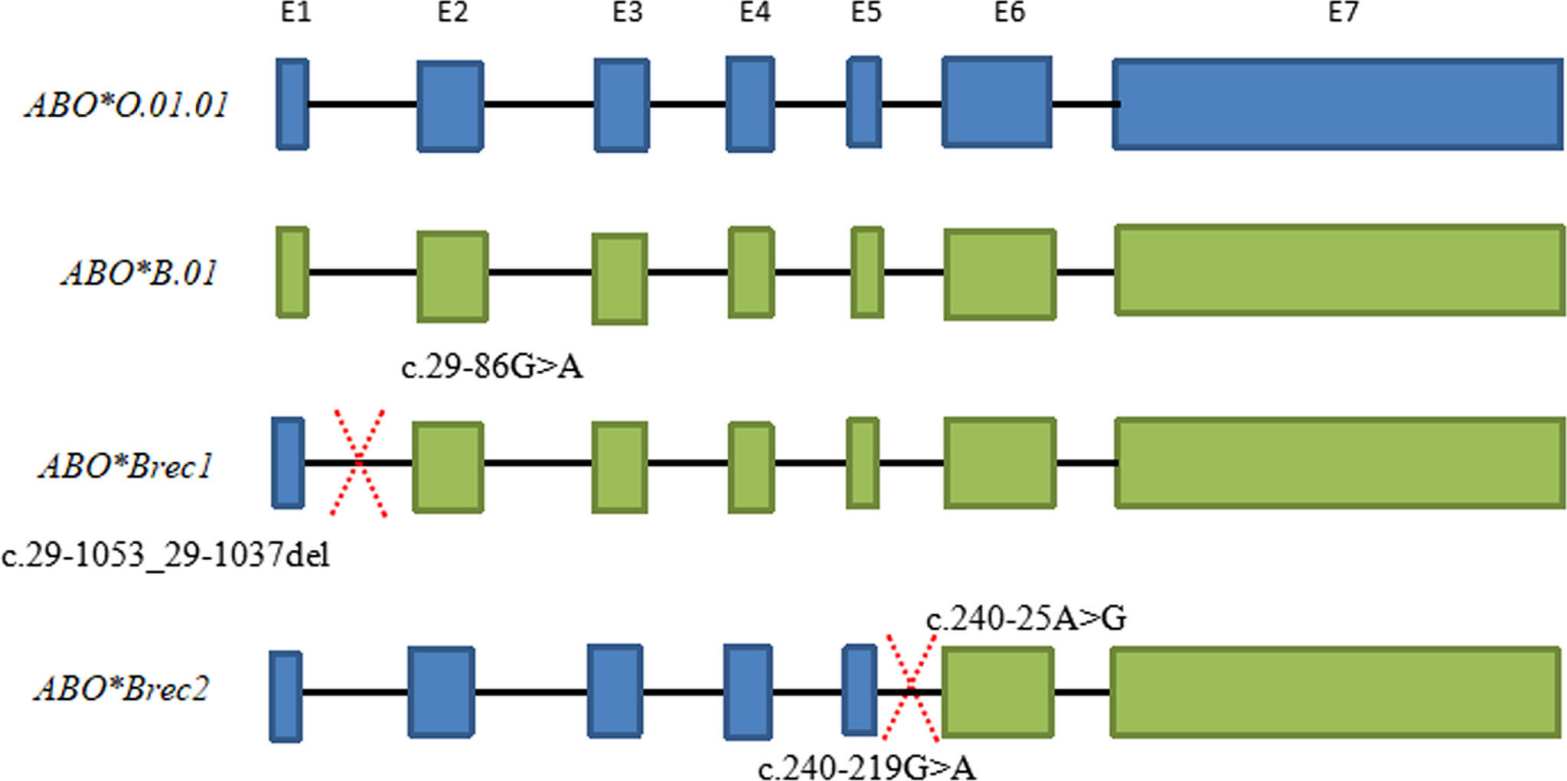
Schematic diagram of *ABO* allele recombination for two specimens. ABO*Brec1 represents the result of allele recombination for the one homozygous individual (ABO*B.01/ABO*B.01). The intron 1 of the ABO*Brec1 allele has the characteristics of the ABO*O.01.01 but sequence from exon 2 to exon 7 has characteristics of the ABO*B.01, and the recombination event maybe happen from c.29-86G>A and c.29-1053_29-1037del. ABO*Brec2 represents the result of allele recombination for the specimen with the ID of 19047. According to the sequence of the ABO*Brec2 allele, the sequence from intron 1 to intron 5 has the characteristics of the ABO*O.01.01 but the sequences from exon 6 to exon 7 have characteristics for ABO*B.01. The recombination event maybe happens from c.240-219G>A to c.240-25A>G.

Six specimens, including one homozygous individual (ABO*B.01/ABO*B.01) and five individuals with ABO subtypes, may exhibit allele recombination. A diagram for the recombination events of these specimens is shown in **Figure 3**. ABO*Brec1 was found in the ABO*B.01/ABO*B.01 homozygous individual. According to the sequences of this sample, the recombination event could be inferred within the span from c.29-86G>A to c.29-1053_29-1037del. c.29-86G>A located in the intron 1 is specific for ABO*B.01 allele, and the sequences seemed to be split into two parts at this specific SNV. The former part was heterozygous for ABO*B.01/ABO*O.01.01 and the latter part was homozygous for ABO*B.01/ABO*B.01, see the detail in **Supplementary S4**. Therefore, one of the B alleles in this individual was found to recombine from ABO*O.01.01 and ABO*B.01.

ABO*Brec2 was found in the sample ID 19047 with the genotype of ABO*B3.10/ABO*O.01.01 and other four individuals with ABO*B3.10 allele. The sequences of exons 6 to 7 were heterozygous for ABO*O.01.01 and ABO*B.01 in the ID 19047, but many SNV sites from intron 1 to intron 5 were homozygous, and the SNV characteristics were related to ABO*O.01.01. The recombination event maybe happens between c.240-219G>A and c.240-25A>G because these two SNVs were specific for the ABO*O.01.01 and ABO*B.01 alleles, respectively (**Table 3**). The heterozygosity of another sample ID 19004 with the genotype of ABO*B3.10/ABO*O.01.01 was almost identical to the sample ID 19047 except for partial sequences located in intron 1. However, in one sample ID 19032 with the genotype of ABO*A.01.02/ABO*B3.10 and two individuals (ID19048 and ID19008) with the genotype of ABO*B3.10/ABO*O.01.02 may also exhibit allele recombination due to some SNVs were homozygous or heterozygous in the intronic region, which was indicated in red in **Supplementary S4**, but the exact region was not well inferred based on the untypical data.

## Discussion

Although NGS methods for *ABO* genotyping have been reported (21–23), most of them cannot analyze the sequence of the full intronic regions. However, in this study, a method for detecting the sequence of the *ABO* gene with coverage from the start codon to the stop codon was successfully established using the NGS platform. Because the genomic full length of the *ABO* gene is over 20 kb with a length of intron 1 over 13 kb, it is difficult to amplify the full-length sequence of the *ABO* gene using one pair of primers. Therefore, two pairs of primers were designed to amplify two overlapping amplicons, which covered the sequence from the start codon to the stop codon of the *ABO* gene. Following sequence analysis using the NGS method, all sequences from the start codon to the stop codon of the *ABO* gene could be successfully assigned. A total of 110 specimens were detected using the NGS method, and the results were the same as those of the PCR-SBT method according to the sequence of the coding region, which suggested that the established NGS method was accurate.

Specific SNVs in intronic regions for various *ABO* alleles have rarely been reported. Here, we first found that some SNV sequences were associated with three common alleles, including ABO*B.01, ABO*O.01.01, and ABO*O.01.02. The ABO*O.01.02 allele had the most specific SNVs among three common alleles and the most specific sites for ABO*O.01.02 allele were located in the intron 4 region. These specific SNVs can be used to design primers for single allele amplification and analysis, to help assign *ABO* alleles and analyze the possibility for allele recombination.

The ABO subtype specimens were also detected using the NGS method, and the sequences of the coding regions were the same as those detected by PCR-SBT. Interestingly, three variants (c.28+5872C>T, c.28+5882C>T, and c.28+5956T>A) in the region of intron 1 were found in five ABO subtype specimens, and no variants were found in the exon or splicing sites. Previous studies have reported that there is an erythroid cell-specific regulatory element (+5.8 kb) in intron 1 of the *ABO* gene, which is responsible for ABO antigen differential expression (25, 31–38). A variant +5904C>T of the RUNX1 site in the erythroid cell-specific regulatory element was identified in our previous study (34), which could decrease antigen B expression to form the B subtype. In this study, c.28+5872C>T (previously +5904C>T) was found in three specimens with the B_3_ phenotype, which was consistent with a previous report (34). c.28+5882C>T around the RUNX1 motif was also found in two specimens with the B_weak_ phenotype, which could decrease antigen expression (37). In addition, a novel variant c.28+5956T>A was first identified in a specimen with the A_3_ phenotype in this study. However, we did not test the function of the variant *in vitro*, and the mechanism by which this variant causes antigen A weakening remains to be further studied.

Allele recombination refers to the exchange of genes controlling different biological traits during sexual reproduction, which does not produce new genes but can produce new genotypes (38–41). Usually, the two DNA strand recombination should be complementary, and the bases near the recombination fracture site should be complementary. The recombination of human leukocyte antigen (HLA)-A/C in two Han families has been reported in our laboratory, which is an important mechanism for HLA evolution (40). Nakajima et al. reported that the recombination of different introns occurred in the *ABO* alleles (38). Therefore, investigating the *ABO* allele recombination is significant to understanding the mechanism of ABO evolution. However, there are few studies on *ABO* allele recombination due to a lack of sequences of the intron regions and specific SNVs of the various alleles. In this study, we found that allele recombination occurred not only in serologically normal specimens but also in specimens with ABO subtypes, and the mechanism of *ABO* allele recombination needs further investigation by pedigree surveys. Cai et al. reported that c.28G>A may cause a B_3_-like subgroup by affecting RNA splicing of the *ABO* gene (42). However, all individuals with c.28G>A in our study had one allele recombination event. Therefore, the molecular mechanism of these five ABO subtypes needs to be further studied.

However, this NGS method cannot be completely used for *ABO* haplotyping, and it is necessary for some specific samples containing new variants to be detected using many different methods, such as cloning technology or allele-specific primer amplification sequencing. In conclusion, an NGS method for sequencing from the start codon to the stop codon of the *ABO* gene has been established. The specific SNV sites for common *ABO* alleles were obtained by detecting homozygous specimens with normal phenotypes. Moreover, two novel *ABO* alleles were identified, and two events of allele recombination were found to occur in the *ABO* gene.

## Data Availability Statement

The datasets presented in this study can be found in online repositories. The names of the repository/repositories and accession number(s) can be found in the article/**Supplementary Material**.

## Ethics Statement

The studies involving human participants were reviewed and approved by The Ethics Committee of the Blood Center of Zhejiang Province. The patients/participants provided their written informed consent to participate in this study.

## Author Contributions

YH and FZ designed experiments. YH and XH performed lab experiments. YH, XH, JZ, and JH analyzed data. YH, FZ, and HH wrote the paper. All authors contributed to the article and approved the submitted version.

## Funding

This work was supported by the National Natural Science Foundation of China (82070195 and 81902137), the Science Research Foundation of Zhejiang Healthy Bureau, China (WKJ-ZJ-1920,2018RC029), and Zhejiang Provincial Program for the Cultivation of High-Level Innovative Health Talents.

## Conflict of Interest

The authors declare that the research was conducted in the absence of any commercial or financial relationships that could be construed as a potential conflict of interest.

## Publisher’s Note

All claims expressed in this article are solely those of the authors and do not necessarily represent those of their affiliated organizations, or those of the publisher, the editors and the reviewers. Any product that may be evaluated in this article, or claim that may be made by its manufacturer, is not guaranteed or endorsed by the publisher.

## Supplementary Material

Supplementary S1 The average of coverage for 94 samples with the same run.

Supplementary S2 The raw data included the nucleotide change of intronic sequence for 47 individuals with homozygotes by the NGS method. Sheet 1 is for the 12 individuals with *ABO*A1.02/ABO*A1.02*, Sheet 2 is for 14 individuals with *ABO*B.01/ABO*B.01*, Sheet 3 is for the 13 individuals with *ABO*O.01.01/ABO*O.01.01*, and Sheet 4 is for eight individuals with *ABO*O.01.02/ABO*O.01.02.* In the “Type” column, SNV represents single-nucleotide variant and MNV represents multi-nucleotide variants.

Supplementary S3 The raw data included the nucleotide change of intronic sequence for five ABO subtypes with no variations in the coding region by the NGS method. Each sheet is named by the sample ID. The nucleotide changes of the intronic sequence were marked in red color.

Supplementary S4 The raw data included the nucleotide changes of the entire *ABO* genomic region for six samples with allele recombination. Each sheet is named by the sample ID. The regions where the recombination events happened were marked in red color.
